# Social factors and the neurobiology of pathogen avoidance

**DOI:** 10.1098/rsbl.2021.0371

**Published:** 2022-02-23

**Authors:** Martin Kavaliers, Klaus-Peter Ossenkopp, Cashmeira-Dove Tyson, Indra R. Bishnoi, Elena Choleris

**Affiliations:** ^1^ Department of Psychology and Neuroscience Program, University of Western Ontario, London, Ontario, Canada N6A 5C1; ^2^ Department of Psychology and Neuroscience Program, University of Guelph, Guelph, Ontario, Canada N1G 2W1

**Keywords:** disgust, parasite, social behaviour, social information, motivation, sex differences

## Abstract

Although the evolutionary causes and consequences of pathogen avoidance have been gaining increasing interest, there has been less attention paid to the proximate neurobiological mechanisms. Animals gauge the infection status of conspecifics and the threat they represent on the basis of various sensory and social cues. Here, we consider the neurobiology of pathogen detection and avoidance from a cognitive, motivational and affective state (disgust) perspective, focusing on the mechanisms associated with activating and directing parasite/pathogen avoidance. Drawing upon studies with laboratory rodents, we briefly discuss aspects of (i) olfactory-mediated recognition and avoidance of infected conspecifics; (ii) relationships between pathogen avoidance and various social factors (e.g. social vigilance, social distancing (approach/avoidance), social salience and social reward); (iii) the roles of various brain regions (in particular the amygdala and insular cortex) and neuromodulators (neurotransmitters, neuropeptides, steroidal hormones and immune components) in the regulation of pathogen avoidance. We propose that understanding the proximate neurobiological mechanisms can provide insights into the ecological and evolutionary consequences of the non-consumptive effects of pathogens and how, when and why females and males engage in pathogen avoidance.

## 1. Introduction

All animals face a threat of exposure to parasites and pathogens. Without even infecting their hosts, parasites can have a range of non-consumptive effects [1–3]. Parasite/pathogen avoidance is a vital component of an animal's ecology and social life. Avoidance responses can range from changes in the social behaviour of infected hosts that affect their interactions with conspecifics (i.e. sickness behaviours [4,5]), to shifts in the behaviour of healthy hosts, which is the focus of this review. These modifications in behaviour can arise either: (i) by directly avoiding or removing visible parasites or (ii) more indirectly, by either avoiding individuals or altering interactions with individuals that may be infected [1,6]. Although used interchangeably here, pathogens and parasites differ in various features, especially the presence or absence of intensity-dependent pathology, respectively (discussed in [7]).

Although there are a variety of studies describing the evolutionary causes and behavioural consequences of parasites and pathogen avoidance (reviewed in [1,8,9]), there have been only limited considerations of the proximate mechanisms that underlie how, when and why pathogen avoidance occurs [1]. Understanding behaviour requires the incorporation of proximate and ultimate levels of analysis and the recognition of cause and function [10–12]. A neurobiological approach incorporates analyses of how individuals sense information, process it and subsequently modify their behaviour and internal states [11,12]. This proximate level of analysis, and the neurobiological mechanisms that it reveals, can provide a framework for investigating and generating hypotheses regarding the non-consumptive (non-lethal) social and non-social behavioural consequences of pathogen avoidance (for extensive recent discussion see [12]).

Parasite avoidance occurs in response to environmental and social factors that are perceived on the basis of various sensory cues that are centrally integrated and lead to context-appropriate behavioural responses. Avoidance can range from a direct avoidance of infected individuals or their cues, to a more general avoidance of interactions with other individuals, which in the human context is now termed ‘social distancing’. Analysis of avoidance involves consideration of cognitive processes, motivation and affective (emotional) states (e.g. disgust and fear). Cognition includes the neurobiological processes involved in the sensory acquisition, processing, retention and use of information associated with parasite threat. Motivation is defined here as the mechanisms responsible for activating, directing and maintaining pathogen avoidance. In the literature on humans, the motivational system for regulating behavioural defences against parasites and pathogens has been termed the ‘behavioural immune system’ [1]. Emotions are considered here as a set of neurobiological responses that occur when the brain detects challenging or rewarding situations [13]. Disgust is considered to be an evolutionarily conserved emotional or emotion-like system whose neurobiological substrates function to detect signs of, and facilitate the expression of pre-emptive and anticipatory avoidance responses to, toxins, contaminants, parasites and pathogens [13–15]. Fear responses may be intertwined with disgust, depending on the nature of the parasite threat (e.g. fear responses to biting flies [1]). Individuals have been described to be navigating through a ‘landscape of disgust’ and the ecological and evolutionary consequences of their responses have been framed in terms of the ‘ecology of disgust’ [2,3]. To function adaptively, pathogen disgust sensitivity should be context-specific and calibrated to the local costs and benefits associated with infection risk and the expression of avoidance behaviours [13]. As such, disgust and its neurobiological underpinnings can provide a framework for addressing the relations between social factors and parasite avoidance.

Although behavioural ecologists have increasingly embraced the relevance of cognitive processes to behaviour [12], they do not often consider the cognitive and emotional mechanisms associated with parasite detection and avoidance. It is important to integrate pathogen avoidance responses and their neurobiological foundations in ecological studies of behaviour. An examination of the underlying neurobiological processes allows one to address the cognitive processes and motivation for, as well as the affective (emotional) states (i.e. disgust) and behavioural consequences associated with, parasite avoidance in both males and females. The latter is especially relevant in that, when present, sex-dependent differences in traits have implications for a number of phenotypes from physiological to behavioural, as well as for susceptibility to various threats, pathogens and disease [16–18]. Indeed, there is mounting evidence for sex differences in infection susceptibility and the expression of disgust-related avoidance responses likely grounded in the different evolutionary pressures on the two sexes [17,18].

Here, we briefly discuss the neurobiology of parasite/pathogen avoidance and how this relates to a broader understanding of the regulation of social behaviour and the utilization of social information. We focus on studies with male and female laboratory rodents and the brain regions and neuromodulatory mechanisms associated with pathogen avoidance that have known roles in the regulation of cognition, motivation, affective states and social behaviour.

## 2. Social information and pathogen avoidance

Social factors influence various aspects of pathogen detection and avoidance [1]. Integral to social behaviour and pathogen avoidance is social information, which can be acquired from the behaviour and products of other individuals through cognitive mechanisms [1,19]. Social cognition includes the perception, acquisition, processing and interpretation of information both about others (social recognition) and from others (social learning). Social information triggers various neural and neuromodulatory processes that allow individuals to assess and quickly respond to potential pathogen threat, while taking into account their own social role, motivation and cognitive abilities. Understanding the neural and neuromodulatory processes that are engaged in individuals of varying sociability, ‘personality’, age and developmental stage, sex and reproductive status can provide insights into their responses to actual and potential pathogen threat.

Social information regarding the condition and nature of potential social partners and mates is used in the determination of pathogen avoidance. This includes establishment of the incentive salience (value) of a potential social/sexual partner and determination of subsequent approach/avoidance behaviours. This involves consideration of the expression and regulation of both preference (bias toward a particular partner or mate) and choosiness (rapid modulation of preference on the basis of immediate information). Social and non-social stimuli that elicit approach behaviour and have rewarding properties are considered positive incentives, while stimuli that elicit avoidance and withdrawal are termed negative incentives [20]. The responsiveness and willingness of an individual to engage in approach/avoidance can be considered to reflect their motivation for social interaction and, or mate acquisition [21]. Both incentive salience and motivation are affected by immediate environmental and social factors (e.g. condition and sexual reproductive status of the individual as well as that of conspecifics), affective and internal states elicited, and their neurobiological consequences.

The impact of various social factors and their neurobiological correlates on behaviour, including that of pathogen avoidance, is important at both the individual and group level [22,23]. Parasites shape host sociality, including group size, territoriality and network structure. While social relationships favour the transmission of social information, they also favour the spread of socially acquired pathogens. These cost–benefits may lead to trade-offs between the acquisition of social information from others and the likelihood of parasite transmission [23]. Understanding the neural and neuromodulatory mechanisms that are engaged during the acquisition of social information regarding parasite/pathogen threat may provide insights into how these trade-offs are expressed and determined (e.g. whether neuromodulatory systems favouring general or specific approach/avoidance are preferentially activated).

Social information about potential pathogen threat that is acquired through sensory and social cues without any direct behavioural interactions can have significant effects on the expression of aversive and avoidance responses. Social responses and interactions that are sensitive to pathogen threat and rely on social cognition that are considered here include: (i) social partner and mate choice and deciding whom to interact with and whom to avoid; (ii) the recognition and avoidance of unfamiliar individuals to exclude potentially infected conspecifics; and (iii) social learning of responses to parasite threat using the responses shown by others.

## 3. Neural substrates of pathogen avoidance

**Figure 1.**
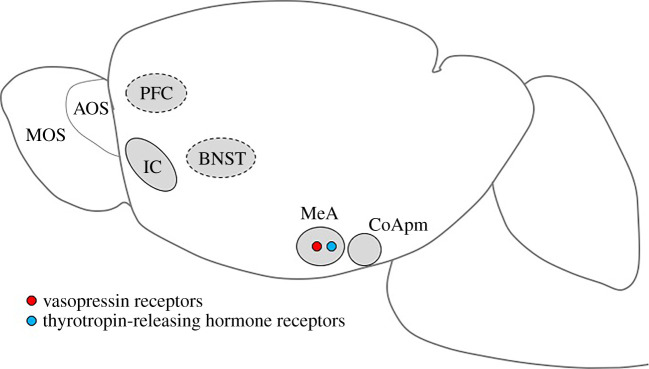
Brain areas involved in pathogen recognition and avoidance by rodents. The red circle represents vasopressin receptors and the blue circle represents TRH receptors. Although several neurochemicals have been identified to be involved in pathogen recognition and avoidance (oxytocin, opioids, serotonin, dopamine, endocannabinoids, glucocorticoids, various immune components, oestrogens and progesterone), their brain sites of action and the receptors mediating those effects remain unknown. In dashed lines are likely candidate regions. MOS = main olfactory bulb; AOS = accessory olfactory system; PFC = pre-frontal cortex; IC = insular cortex; BNST = bed nucleus of the stria terminalis; MeA = medial amygdala; CoApm = posteromedial nucleus of the cortical-amygdala.

**Figure 2.**
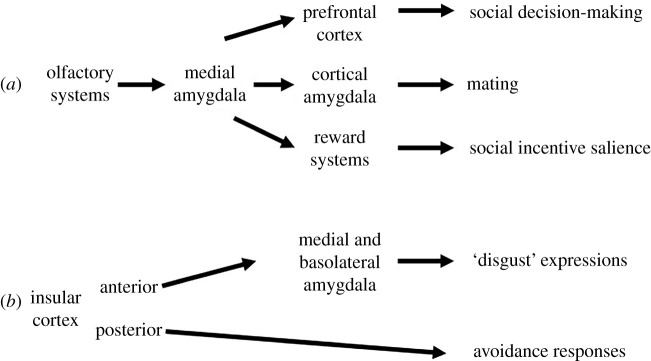
Neural pathways are involved in the mediation of social decision-making, mating, social incentive salience, ‘disgust’ expressions and avoidance responses. We propose that these same pathways mediate various aspects of pathogen recognition and avoidance in rodents. Additional systems that are part of the social decision-making network are also likely to be involved.

Pathogen detection and avoidance involve a variety of central brain regions, including the evolutionarily conserved social decision-making network (as illustrated for mammals in figures 1 and 2). This encompasses the social brain and reward network [24,25]. The social decision-making network incorporates a number of brain areas, including the amygdala, insular cortex, pre-frontal cortex and various reward and olfactory regions that are relevant to threat detection and pathogen avoidance. These areas are either directly or indirectly involved with the detection and processing of the valence and salience of social and sensory cues that relate to pathogen avoidance [26–28]. Selective activation or inhibition of specific brain regions and circuits can determine the nature of the behavioural responses displayed towards other individuals (e.g. investigate (approach/avoid), decide to mate, aggression, surrender and flee). Understanding if, how, when and why different brain areas are activated (e.g. how sex and reproductive status, age, condition and experience impact on neural activity) can aid in elucidating an individual's motivation and likely behavioural responses to pathogen threat under various social and environmental conditions.

### (a) Sensory cues and pathogen avoidance—olfactory systems

Identifying stimuli indicative of pathogen threat and responding to the characteristics and information associated with these stimuli is integral to pathogen avoidance. What sensory cues individuals should use depends on the availability, social salience (importance), valence (positive or negative), costs, timeliness and redundancy of those cues. A cue is considered here as a feature of the emitter that communicates something to the receiver as an incidental by-product, as opposed to a signal that has a direct communicative function.

In many species, chemical information and odours are especially important for the recognition and assessment of condition and infection status (reviewed in [1]). Odour-based recognition of infection status is important for determining whether or not to engage in subsequent behavioural interactions (i.e. approach/avoidance) and the nature of those social interactions (e.g. affiliation, aggression, mate choice and sexual responses) [1]. Odours, which are particularly salient cues of infection status and parasite threat in rodents, are a complex mixture of volatile and non-volatile peptides, proteins and steroids [29,30]. Volatile components permit individual assessment from a distance, while non-volatile components necessitate more intimate contact with the odour source and a heightened risk of possible infection [29,31]. Pathogens can influence odour-based avoidance through modifications in the quality and quantity of urine and faecal components, along with chemical signals from other sources (e.g. tears, saliva, preputial glands, external and internal microbiome components). Various pathogenic substances (bacteria and their components (lipopolysaccharide (LPS), the outer cell wall of Gram-negative bacteria), viral mimics, nematode parasites) produce different volatile odour constituents in rodents [32]. Also, gut microbiota produce a variety of metabolites that affect social odours and are influenced by infection status [33]. In addition, learned associations to the odours of either parasitized individuals or toxin-induced sickness can elicit disgust responses (facial expressions and emetic-like responses in the case of toxins) and behavioural avoidance [34–36]. Understanding the mechanisms associated with the sensory perception and neural integration of odour cues can aid in determining the non-consumptive behavioural responses to pathogen threat.

In rodents, odour-mediated recognition involves detection mechanisms and receptors in two complementary, though distinct, olfactory systems—the main olfactory system (MOS) and accessory olfactory system (AOS) (figure 1). The MOS is involved in social odour detection at a distance, while the AOS requires closer contact. The MOS and AOS involve neurons in the main olfactory epithelium and olfactory epithelium in the vomeronasal organ, respectively [29], with outputs that are conveyed to multiple brain regions [31,37–39]. In mice, vomeronasal sensory neurons (VSNrs) detect non-volatile chemical stimuli indicative of infection and genetic compatibility [40]. The VSNrs include formyl peptide chemoreceptors that respond to specific bacterial cues, including bacterial toxins and LPS [41].

Odour-based recognition and condition assessment involve at least two highly polymorphic gene complexes, the major histocompatibility complex (MHC) and the major urinary protein (MUP) cluster [29,30]. The MHC class I gene complex is directly related to condition through its linkage to immune function, while non-volatile MUPs are carriers of volatile ligands that provide information about condition and individual identity [29]. For example, a male-specific MUP, darcin, elicits innate attraction, allowing female mice to recognize and assess individual males on the basis of their odours [42]. Male mice treated with LPS had reduced levels of darcin in their urine and were avoided by females [43]. However, females also avoided the urinary odours of nematode-infected males whose darcin levels were unchanged, supporting the involvement of other constituents of male odours [1]. In addition, males avoid the odours of, and mating with, LPS-treated females [44]. The responses to darcin may be dependent on a female's internal state, which is affected by other odour components and sensory/social cues associated with infection threat. There are likely a variety of species-, sex-, infection- and context-dependent odour constituents that affect pathogen detection and avoidance. For example, sharing an environment with a sick or infected conspecific can affect the odour of a healthy mouse and subsequent responses to that individual [1,45]. Similarly, the stage of infection and risk of pathogen transmission can influence avoidance. For example, the odours of males at a non-transmittable stage of infection with a coccidian parasite failed to elicit any behavioural avoidance by female mice [1]. Thus, knowing what chemical cues are produced, how they are perceived (volatile or non-volatile; infectious versus non-infectious), and how they may influence the odours of others, can aid in the prediction of the detection of, and subsequent responses to, pathogen threat.

### (b) Nociceptive and pain responses

Various defence mechanisms underpin the survival of animals when confronted with a threat. Changes in pain sensitivity are integral to this [40,46]. Across species various stressors and threats have been shown to affect individual nociceptive (pain or pain-like) sensitivity. In addition to influencing approach/avoidance responses, exposure to the odours of threatening individuals also adaptively modulates nociceptive sensitivity. Odours of infected individuals elicited an acute decrease in acute pain sensitivity (antinociception, analgesia; measured in rodents as the time to respond to a thermal stimulus) followed by a delayed increase in pain sensitivity (hyperalgesia) [1,47]. These shifts in pain sensitivity and their neurobiological correlates can enhance avoidance responses, shifting the motivational and affective state and enhancing the salience of infected individuals. The initial analgesic responses and their anxiety/fearfulness/stress-associated correlates can facilitate the expression of defensive behaviours and active avoidance responses (e.g. fleeing and burying to avoid biting flies [48]), while the later increased pain sensitivity promotes augmented vigilance and enhanced sensitivity to threats [1,49]. In addition, there are sex differences in the magnitude and neurobiological regulation of nociceptive responses that need to be incorporated when examining responses to pathogens and other threats [40]. Although generally not considered in behavioural ecology, changes in nociceptive and pain sensitivity are factors that merit further attention, especially in relation to predator and pathogen avoidance.

#### (i) Neural substrates—amygdala

As a ‘salience detector’, the amygdala is critical to the identification and decoding of social stimuli and elicitation of context-appropriate social responses (figures 1 and 2). Olfactory inputs from the AOS and MOS are conveyed to the medial amygdala, where non-volatile and volatile information is integrated [31,37,38]. This information is relayed to the pre-frontal cortex, which is essential for social decision-making. Results of imaging studies with humans showed that pictures of sick LPS-treated individuals led to decreased activity in the ventromedial pre-frontal cortex and the initiation of avoidance [50]. In addition, there are projections from the amygdala to the reward systems and other brain regions where the incentive salience and social reward value of a conspecific and the likelihood of subsequent approach/avoidance are determined [24,25]. Parasitized individuals and their cues can be considered to be of lower reward value and reduced incentive salience, leading to a reduced interest in, and avoidance of, them. Whether or not pathogen exposure-elicited changes in the activity of neural regions associated with salience detection may also impact on responses to other rewarding factors (e.g. dietary components and habitats) remains to be determined.

Results of recent studies have shown that the optogenetic activation of the dorsomedial nucleus of the cortical-amygdala (CoApm), a region with olfactory inputs and outputs to the medial amygdala, inhibits males from mating with LPS-treated female mice [44]. In addition, the odour of an LPS-treated female is sufficient to activate the CoApm and, thereby, inhibit male mating even with a healthy female. Interestingly, this activation does not appear to alter sociability towards sick same-sex individuals, suggesting sex- and context-specific responses.

#### (ii) Neural substrates—insular cortex

The insular cortex is integral to the expression of affective states, especially that of disgust and disgust-like responses. The insular cortex is involved in the processing of aversive sensory stimuli and internal states and exerts ‘top down’ control on various avoidance/aversive behaviours through intimate connections with components of the social brain network and reward regions [51].

The insular cortex is directly involved in the modulation of pathogen avoidance and the expression of disgust. Pharmacological inactivation of the posterior insular cortex eliminated the avoidance responses that male and female rats displayed to adult rats treated with a viral mimetic [52]. In humans, results of imaging studies showed that individuals exposed to LPS-treated subjects and their odours, which elicited avoidance and disgust responses, displayed increased activity in the medial amygdala and insular cortex [53]. In mice, toxin (lithium chloride)-elicited facial expressions indicative of disgust were associated with augmented neuronal activity in the anterior insular cortex. In the absence of toxin, these disgust expressions could be elicited by optogenetic manipulation of the activity of the insula [54]. The expression of these disgust responses involved neural pathways from the anterior insula to the medial and basolateral amygdala [55]. How various social and environmental factors modulate the functioning of the insula, and whether or not there are sex differences in responses, remain to be determined.

Social decision-making and its underlying neural substrates enable animals to respond to their social environment with flexible, context-appropriate, behaviours [1,25]. Recognizing and evaluating cues from others, and using that information to choose whom to either approach or avoid, is vital to successfully navigating social situations [56]. Pathogen detection and avoidance likely involve coordinated activity across various central brain regions and the integration of sensory inputs, salience and reward values to elicit context-appropriate behavioural responses (figure 2). Knowing which brain regions and circuits are activated by pathogen/parasite threat will allow a fuller understanding of avoidance and other behavioural responses elicited.

## 4. Neuromodulatory substrates of pathogen avoidance

Pathogen avoidance further involves various neurotransmitters, sex steroid hormones, other steroid hormones (e.g. glucocorticoids), nonapeptide systems (oxytocin, arginine–vasopressin (AVP) and their receptors), other neuropeptides and their receptors, as well as immune system and microbiome components [1,56,57]. These candidate neuromodulatory systems, through their actions in various brain regions and networks, allow individuals to rapidly evaluate and respond to information associated with pathogen threat.

### (a) Oxytocin

Oxytocin has been implicated in a number of social domains, including processing of salient social stimuli, social recognition, social interactions (including social vigilance, social distancing and social approach/avoidance), social learning and social memory (e.g. [13,27,28,57–62]). Oxytocin receptors (OTRs) are proposed to modulate the activity of the social decision-making network and related cortical structures that are associated with the encoding and processing of positive and negative inputs and the incentive salience of social and sensory cues [61]. Oxytocin affects the activity of the insular cortex, with inhibition of the insular cortex or blockade of insular OTRs disrupting social affective behaviour in rats [51]. In addition, oxytocin and OTRs in the medial amygdala are important for social recognition [63,64], and likely the responses to social cues associated with infected individuals.

Oxytocin enhances the salience of both positive and negative social stimuli (Social Salience Hypothesis [62]). Oxytocin mediates both approach and avoidance responses to positive and negative salient social information, respectively. Oxytocin acting in the reward areas facilitates social approach, while aversive contexts, which elicit social vigilance and avoidance, involve other motivationally associated regions [60,61]. The type of behaviour observed depends on the nature of the social stimulus, social context and sex of the individual [65,66], with oxytocin enhancing social avoidance and aversive response to threatening social stimuli to a greater extent in females than in males [67]. In this regard, oestrogens facilitate social recognition by regulating the action of oxytocin action in the hypothalamus and that of the OTR in the medial amygdala [63,64].

Oxytocin has been shown to be directly involved in the recognition and avoidance of infected individuals and their odours. Systemic administration of an OTR antagonist attenuated the avoidance responses of male and female rats to the odours of LPS-treated conspecifics [1,68,69]. Female mice either with deletions of the oxytocin gene (oxytocin knockout (OTKO) mice), or systemically treated with a selective OTR antagonist, were also impaired in their avoidance of, and aversive responses (including analgesic responses) to, the urinary odours of nematode-parasitized and LPS-treated individuals [70–72]. However, their responses to either predator odour or the odour of a restraint-stressed conspecific were not affected.

Oxytocin is also implicated in the expression of toxin-elicited disgust. Rats display conditioned disgust responses (anticipatory nausea—gaping indicative of emesis) to a social context associated with a toxin. Systemic treatment with an OTR antagonist attenuated the expression of this socially determined anticipatory disgust [73]. This suggests that oxytocin may be associated with the broad expression of disgust. As such, social factors that can influence oxytocin release and or functioning may impact on disgust sensitivity and the expression of pathogen avoidance behaviours.

Social learning also influences responses to pathogens in an oxytocin-dependent manner. Female mate choice copying is a type of social learning that occurs when a female's likelihood of mating with a male is influenced by the apparent direct or indirect choice of another female [74]. The avoidance responses normally displayed by female mice to a parasitized male were markedly reduced when the odours of an oestrous female were associated with that particular male. The olfactory-related interest displayed by another female likely enhances the sexual incentive salience of the infected male. This again illustrates the influence of social and mate-related factors on pathogen avoidance. It also shows the need to incorporate cognitive factors and prior experience when examining pathogen avoidance.

OTKO female mice and females systemically treated with an OTR antagonist were impaired in this social learning and did not exhibit pathogen-related mate choice copying [71]. Females also avoid males that are associated with the odours of infected males. Systemic treatment with an OTR antagonist similarly blocked the copying of the avoidance response [72]. Oxytocin at the level of the medial amygdala has also been directly implicated in the mediation of social learning of fear responses in mice [75]. This raises the possibility of oxytocin being involved in the mediation of the socially learned fear and avoidance responses to ectoparasites (biting flies) displayed by mice [48]. As such, natural social and environmental factors that affect oxytocin release and levels could influence pathogen avoidance responses.

The actual and potential threats posed by individuals from out-groups (unfamiliar individuals, strangers) are also important determinants of social behaviour [1]. Out-group individuals can present a risk of pathogen exposure and threats to territory, resources and offspring. These threats can affect social approach and/or avoidance and an individual's motivation to interact with in-group members (familiar individuals). Exposure to, or the presence of, unfamiliar individuals can promote a greater wariness of potential parasite threat. Out-group threats are common in social species. However, it should also be remembered that social animals typically exhibit obligate dispersion that would necessitate interactions with unknown conspecifics.

Oxytocin is involved in the modulation of the augmented responses to familiar individuals displayed after exposure to pathogen threat (in-group (familiar) bias and out-group (unfamiliar) avoidance), as well as the enhanced sensitivity to pathogen threat seen after exposure to strangers [59]. In humans, intranasal oxytocin augmented positive responses to members of in-groups and negative responses to out-group members, promoting intergroup discrimination [59]. Oxytocin also amplified intergroup recognition and discrimination, leading to enhanced social vigilance/anxiety towards out-groups. However, not all studies show a consistent effect of oxytocin, indicating that additional neuromodulatory factors are associated with pathogen threat-elicited group biases.

The presence of pathogen threat also influences responses to unfamiliar individuals in mice [72]. Systemic treatment with an OTR antagonist attenuated the aversive and avoidance responses to unfamiliar males displayed by female mice after exposure to an infected individual. Similarly, an OTR antagonist blocked the augmented responses to familiar males evident after exposure to pathogen threat [72]. Oxytocin likely permits the display of behavioural avoidance responses to previously rewarding unfamiliar individuals through its modulation of cortical-amygdala circuits [28].

OTR activation appears to inhibit social approach by increasing social vigilance towards unfamiliar, and possibly threatening, individuals and their cues [67]. Social approach and avoidance involve different neural circuits and neurotransmitters. Oxytocin acting in reward areas facilitates social approach and reward, while aversive contexts, which elicit social vigilance, recruit the bed nucleus of the stria terminalis, which is a target for olfactory information [65,66].

It is further suggested that stress may link the oxytocin circuits between sociability, social approach and social vigilance [67]. This raises a mechanism whereby immediate pathogen threat could elicit social vigilance and social distancing, minimizing social attraction and interactions. Together, these observations show that oxytocin conveys social salience in a variety of sensory modalities, leading to a broadly based pathogen detection and expression of disgust and avoidance responses. It also suggests that oxytocin may be involved in the modulation of social networks and social interactions, both between and within groups of familiar males and females. These observations further indicate the need to consider prior familiarity and social context (i.e. presence of familiar/unfamiliar individuals) when examining pathogen avoidance and its neurobiological underpinnings.

### (b) Arginine–vasopressin

There is also evidence for the involvement of arginine–vasopressin (AVP) in the mediation of social recognition, particularly in males [58]. The AVP system consists of a series of highly sexually dimorphic brain nuclei, including the bed nucleus of the stria terminalis, the amygdala and the lateral septum [76]. Further, there is evidence suggesting that testosterone and its metabolites, including oestradiol, influence social recognition by modulating the activity of AVP receptors.

Male rats avoided social interactions with LPS-treated individuals, with this effect blocked by infusion of an AVP receptor antagonist into the medial amygdala [68]. Interestingly, an OTR antagonist here also blocked approach to a healthy conspecific. Whether or not these effects also involve interactions with oxytocin-associated mechanisms remains to be determined. The extent of the effects of vasopressin in rats was dependent on the age and sex of the individuals providing, as well as receiving, the odours [68]. This further reinforces the need to incorporate sex and social context when examining the expression and regulation of pathogen avoidance.

### (c) Opioids and neurotransmitters

A broad range of neurotransmitters and other signalling molecules are involved in the modulation of social behaviour (reviewed in [77]). Here, we consider only those systems that, to date, likely have some association with pathogen avoidance.

Opioid systems are involved in the regulation of social behaviours and social reward [78]. Opioids can modulate the socio-sexual incentive value of conspecifics and the likelihood of social interactions. It has been proposed that opioids are differentially released during naturally occurring behaviours inducing a reward state, augmenting incentive salience and increasing the probability of approach and social interaction. Alterations in opioid activity following systemic administration of antagonists have been linked to shifts in mate choice and the avoidance of protozoan-infected males by female mice [79]. These effects of opioids may be either direct or indirect through changes in oxytocin function [61]. This raises the need to consider the impact of behaviours that influence endogenous opioid release (e.g. sexual arousal, rewarding food) when considering pathogen avoidance.

In addition to the opioid systems, biogenic amines (dopamine and serotonin) have been linked to pathogen avoidance. Both dopamine and serotonin have been associated with the salience-related effects of oxytocin [61]. Systemic administration of a serotonin receptor antagonist to female mice attenuated their avoidance of the odours of parasitized males [80]. In addition, manipulations of serotonin activity, either peripherally or in the insular cortex, attenuated the display of toxin-elicited disgust [81].

Dopamine has a major role in the expression of social/sexual behaviour. According to the Incentive Salience Hypothesis, dopamine assigns value or salience to objects or actions (e.g. approach to conspecifics) [82]. Reductions in dopamine activity reward system were associated with the display of toxin disgust [75]. Although the roles of dopamine in the modulation of pathogen avoidance remain to be determined, it is likely that rewarding social stimuli (i.e. sexual cues) that augment dopamine release will affect the degree of disgust elicited and pathogen avoidance displayed. This further indicates the need to consider how the presence of rewarding stimuli may modulate pathogen avoidance.

Endocannabinoid involvement in the expression of pathogen disgust should also be considered. Endocannabinoids have been associated with the modulation of a range of neurotransmitter systems, including those that may be related to pathogen avoidance. Endocannabinoids have been implicated in the modulation of the expression of toxin-elicited disgust and anticipatory nausea [83]. Also, endocannabinoid signalling is involved in the mediation of the effects of oxytocin [84].

### (d) Thyrotropin-releasing hormone

Recent studies examining the attenuated mating responses of male mice to LPS-treated females revealed a role for thyrotropin-releasing hormone (TRH) [44]. It was shown that exposure of males to LPS-treated females (or their odours) led to the activation of the medial amygdala and expression of behavioural avoidance. TRH-dependent input to the medial amygdala was necessary for the behavioural avoidance of LPS-treated females by males. This further illustrates the important role of odours, the amygdala and various neuropeptides in the modulation of pathogen avoidance.

### (e) Glucocorticoids

Exposure to infected individuals or their odour cues also results in stress responses and the elevation of glucocorticoids such as corticosterone [1,71]. Exogenous systemic corticosterone has been shown to attenuate sexual motivation, that is, interest in and the incentive salience of, social and sexual stimuli [85]. This raises the possibility that acute elevations in corticosterone and other stress response components either directly, or indirectly through their impact on other neurobiological mechanisms, could contribute to the reduced interest in, and responses to, infected individuals. Indeed, it has been proposed that stress responses enhance oxytocin-modulated social vigilance [60,61]. As such, stress responses and elevations in glucocorticoid exhibited under natural conditions could significantly influence pathogen avoidance-related behaviours. This suggests that the presence of various other threats or stressors (e.g. predators and their cues, dominant conspecifics) could influence pathogen avoidance-associated behaviours. Whether or not both acute and chronic stress and glucocorticoid release would elicit similar effects remains to be determined.

### (f) Immune system

The immune system is a critical mediator of responses to infection and sickness and is likely involved in the mediation of responses to the threat of infection. Infection and sickness behaviours are associated with immune responses (see recent reviews [17,18,86]). However, the nature of the changes in social behaviour is context- and social partner-specific. Further, there are sex differences in immune responses to infectious agents, with females typically showing amplified immune responses [86]. The pre-frontal cortex, amygdala and reward systems appear to be important substrates for immune-driven changes in social behaviour [86]. Also, the insular cortex is involved in the encoding and retrieving of specific immune responses [87].

Exposure to a sick conspecific is thought to prime the subject's immune system so that it can better respond to a potential attack by the same pathogen. Results of human studies have revealed that viewing images associated with disease and sick individuals or rotting food can stimulate immune responses [88]. Exposure to infection threat (LPS-treated individuals) was similarly suggested to modestly affect aspects of immune activity in mice [89]. Results of studies with canaries revealed that visual exposure to individuals infected with an avian bacterial pathogen altered components of immune responses in healthy individuals [90]. The behavioural consequences of this remain to be determined, though it was speculated that depending on the context, individuals facing pathogen threat may invest in immune activation rather than behavioural avoidance. The specific neural correlates associated with the increased immune responses seen after exposure to an infected individual remain to be determined.

### (g) Oestrogens

Oestrogens are also involved in the regulation of affective states and responses to social information [13,91,92]. Oestrogens exert their actions through a number of widespread oestrogen receptors (ERs): ERα, ERβ and the G protein coupled GPER1, which can mediate both rapid non-genomic and longer-lasting classical genomic effects [93]. ERα and ERβ have been implicated in the expression of pathogen detection and avoidance. ERα and ERβ gene-deleted mice displayed minimal avoidance of, and aversive responses to, the odours of parasitized individuals [93,94]. These effects of ERs on pathogen detection and avoidance may be related to their association with oxytocin as ERβ and GPER are involved in the regulation of the synthesis and release of oxytocin [76,93]. All three oestrogen receptors are expressed in the medial amygdala, where they enhance social recognition [95] and are associated with the functioning of the OTR [63,92]. It is conceivable that such interactive mechanisms between oxytocin and ERs are involved in the expression of pathogen and toxin disgust. This reinforces the need to consider sex differences and reproductive status when investigating pathogen avoidance.

### (h) Progesterone

Progesterone has also been suggested to be involved in the expression of disgust. Progesterone has been implicated in the enhancement of pathogen and toxin avoidance in women (Compensatory Prophylaxis Hypothesis [96,97]). In this proposal, progesterone-linked immunosuppression was associated with an increased detection of, and aversive responses to, pathogen cues. In women, disgust responses have been reported to vary across the menstrual cycle, being at their greatest when progesterone is elevated (reviewed in [97]). However, consistent negative findings have also been reported [98,99]. Recently, systemic administration of progesterone to female mice was suggested to rapidly enhance their avoidance of infected males, though social recognition *per se* was also affected [100]. How the possible effects of progesterone may relate to oestrogen and oxytocin involvement in the modulation of pathogen avoidance remains to be determined. Together these findings support the need to consider sex steroid levels and their modes of action when examining pathogen avoidance.

## 5. Conclusion

Parasite and pathogen avoidance is a crucial component of an animal's ecology and social life. Integral to this is determining what motivates and modulates pathogen avoidance and, thus, predicting how it may be influenced by various social and environmental factors. This allows one to generate hypotheses as to what other behavioural and related functions may be affected by pathogen threat and under what environmental and social conditions [11,12]. What are the neural circuits and neuromodulatory mechanisms for activating, directing and maintaining avoidance? What are the relative effects of neuromodulation on motivational and affective systems versus on the circuits responsible for the perception and processing of sensory stimuli? How do these neurobiological mechanisms relate to various other behavioural (social and sexual) functions?

Understanding the neurobiology of pathogen avoidance allows predictions to be made regarding the diverse non-consumptive behavioural and non-behavioural effects evident in different host–parasite systems [101]. It also allows individual differences in response and ‘personality’ to be considered. For example, understanding which neuroendocrine mechanisms are engaged can provide insights regarding the effects of different forms of parasite/pathogen threat on the major personality traits, namely sociability, aggressiveness, activity, exploration and boldness [102]. Conversely, understanding the underlying neurobiology could give insights into how individual differences and personality traits may affect pathogen disgust sensitivity and the expression of pathogen avoidance under different social and environmental conditions. In a like fashion, understanding the neural/neuroendocrine mechanisms allows predictions to be made regarding the influence of sex, age and developmental stage, social and reproductive status on pathogen avoidance. For example, human and non-human adolescents are suggested to have reduced insular activity and augmented reward and dopamine activity, suggesting heightened risk-taking and lowered disgust sensitivity and pathogen avoidance [103]. Similarly, there are age-related shifts in the activity of the pre-frontal cortex, a brain region that has been implicated in pathogen avoidance as well as a range of other functions, including responses to food rewards [50].

What sensory cues are important also requires further consideration. Although we have focused on odours and rodents, the roles of social information provided by multi-modal sensory cues need to be considered. Understanding the underlying neurobiological mechanism allows the influences of ongoing and prior social and environmental cues and factors (e.g. novel environmental stressors and their effects on brain circuits) on the expression of pathogen avoidance and disgust to be interpreted. In turn, the neuromodulatory mechanism influenced by these factors can aid in generating predictions regarding other potential responses to pathogens. This is particularly crucial when comparing the behavioural responses of males and females of different reproductive conditions and sex steroid levels (e.g. fluctuations in oestrogen and testosterone and their effects on parasite avoidance).

We have highlighted the intimate relationship between parasite/pathogen avoidance and the social landscape. Various aspects of social behaviour are affected by, and themselves influence, pathogen detection and avoidance (e.g. social valuation, social salience and social distancing (approach/avoidance). These actions engage a variety of neural and neuromodulatory systems whose functions in pathogen avoidance are only beginning to be determined. Technological advances in neuroscience, including recent advances in genomics (e.g. examination of transcriptional signatures elicited by threats), present new opportunities for assessing brain–behaviour relationships and the expression of pathogen avoidance.

The studies described here have been almost exclusively conducted with rats and mice under laboratory conditions. Investigations of the proximate neural and neuromodulatory mechanisms associated with pathogen avoidance under naturalistic and semi-naturalistic environments, such as those examining sickness behaviour and sexual and neuroendocrine responses, are needed [8,104,105]. A related question is how might a changing environment impact on neuromodulatory mechanisms and pathogen avoidance? Endocrine disrupters, pollutants and climate change may alter both hormonal levels (e.g. sex steroids and glucocorticoids) and sensory perception and integration. Studies across taxa and social systems (e.g. polygamous and monogamous social systems that differ in in the expression of oxytocin and vasopressin [57]) and levels of pathogen threat are necessary to more fully delineate the proximate and ultimate mechanisms underlying pathogen detection and avoidance.

## Data accessibility

This article has no additional data.
